# Pressure Stimuli Improve the Proliferation of Wharton’s Jelly-Derived Mesenchymal Stem Cells under Hypoxic Culture Conditions

**DOI:** 10.3390/ijms21197092

**Published:** 2020-09-25

**Authors:** Sang Eon Park, Hyeongseop Kim, Soojin Kwon, Suk-joo Choi, Soo-young Oh, Gyu Ha Ryu, Hong Bae Jeon, Jong Wook Chang

**Affiliations:** 1 Stem Cell Institute, ENCell Co. Ltd., Seoul 06072, Korea; earnie.park@encellinc.com (S.E.P.); hyeongseop09@encellinc.com (H.K.); soojink@encellinc.com (S.K.); 2 Stem Cell & Regenerative Medicine Institute, Samsung Medical Center, Seoul 06351, Korea; 3 Department of Obstetrics and Gynecology, Samsung Medical Center, Seoul 06351, Korea; drmaxmix.choi@samsung.com (S.-j.C.); ohsymd.oh@samsung.com (S.-y.O.); 4 Department of Medical Device Management and Research, SAIHST, Sungkyunkwan University School of Medicine, Seoul 06351, Korea; gyuha.ryu@samsung.com; 5 The Office of R&D Strategy & Planning, Samsung Medical Center, Seoul 06351, Korea

**Keywords:** hypoxia, physical force, pressure, proliferation, Wharton’s jelly-derived mesenchymal stem cells

## Abstract

Mesenchymal stem cells (MSCs) are safe, and they have good therapeutic efficacy through their paracrine action. However, long-term culture to produce sufficient MSCs for clinical use can result in side-effects, such as an inevitable senescence and the reduction of the therapeutic efficacy of the MSCs. In order to overcome this, the primary culture conditions of the MSCs can be modified to simulate the stem cells’ niche environment, resulting in accelerated proliferation, the achievement of the target production yield at earlier passages, and the improvement of the therapeutic efficacy. We exposed Wharton’s jelly-derived MSCs (WJ-MSCs) to pressure stimuli during the primary culture step. In order to evaluate the proliferation, stemness, and therapeutic efficacy of WJ-MSCs, image, genetic, and Western blot analyses were carried out. Compared with standard incubation culture conditions, the cell proliferation was significantly improved when the WJ-MSCs were exposed to pressure stimuli. However, the therapeutic efficacy (the promotion of cell proliferation and anti-apoptotic effects) and the stemness of the WJ-MSCs was maintained, regardless of the culture conditions. Exposure to pressure stimuli is a simple and efficient way to improve WJ-MSC proliferation without causing changes in stemness and therapeutic efficacy. In this way, clinical-grade WJ-MSCs can be produced rapidly and used for therapeutic applications.

## 1. Introduction

Mesenchymal stem cells (MSCs) were first reported in 1960 [1]. These cells attach themselves to plastic culture flasks in a similar form to fibroblasts, and are defined as adult stem cells with adipogenic, osteogenic, and chondrogenic properties. MSCs are defined by criteria established by the International Society for Cell and Gene Therapy: (1) they are plastic adherent; (2) they have the potential for three-lineage differentiation (adipogenic, osteogenic, chondrogenic); (3) they have positive surface markers (CD44, CD73, CD90, CD105, and CD166) and negative surface markers (CD11b, CD19, CD14, CD45, and HLA-DR) [2].

MSCs are considered to be a promising treatment for rare and intractable diseases, as these cells have the immune privilege (they do not cause immune reactions when transplanted into a person), and have demonstrated therapeutic efficacy in various diseases through their paracrine action [3,4]. Such secretions of proteins—which maintain patient homeostasis, remove causative substances, and regenerate damaged tissue—are the main therapeutic mechanism of MSCs [5,6]. Consequently, the dosage of MSCs needs to be relatively high compared to other stem cell therapies.

The number of stem cells currently used in clinical trials ranges from 10^7^ to 10^9^ cells [7]. Therefore, producing sufficient numbers of MSCs is very important in MSC therapy [8,9]. In order to produce a sufficient number of MSCs from a single donor, continuous, long-term cultures are needed. However, this long-term culture inevitably induces MSC senescence, resulting in the reduction of their multipotency and therapeutic efficacy [10,11,12]. Therefore, the promotion of cell proliferation while retaining MSC stemness in the early stages of the primary cultivation is crucial [13]. In order to address this issue, attempts have been made to simulate the stem cell niche in vitro.

One source of MSCs is Wharton’s jelly, which is found in the umbilical cord. Umbilical cord blood, tissue, and stem cells reside in a hypoxic state [14]. Previous studies have reported that the exposure of MSCs to hypoxic conditions increased their proliferation and decreased their doubling time [15,16,17]. In the umbilical cord, the amnion membrane wraps tightly around the umbilical cord [14]. This compresses Wharton′s jelly, suggesting that WJ-MSC exists under much higher pressure than normal atmospheric pressure [14,18,19]. Based on this rationale, we hypothesized that exposure to hypoxia and high-pressure conditions could mimic the de novo environment of the umbilical cord. We speculated that applying physical forces, particularly pressure, during manufacturing would enhance the yields and proliferation without promoting the senescence of WJ-MSCs.

Thus, we here evaluated the influence of pressure stimuli on the proliferation, stemness, and therapeutic efficacy of WJ-MSCs.

## 2. Results

### 2.1. Pressure Stimuli Promote WJ-MSC Yield

**Figure 1:**
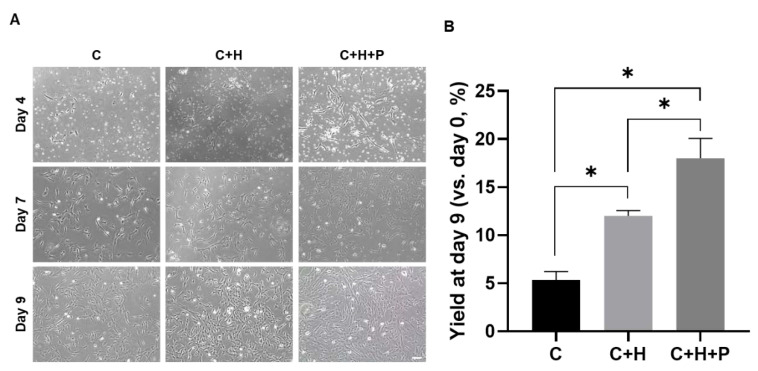
Confirmation of the yield of Wharton’s jelly-derived mesenchymal stem cells (WJ-MSCs) under high-pressure conditions. (**A**) Observation of star-shaped cells by optical microscopy on the 4th, 9th, and 9th days of the initial culture of WJ-MSCs. The WJ-MSCs were cultured under control, 5% hypoxia, or 5% hypoxia with 2.0 PSI pressure conditions. Scale bar = 100 µm. (**B**) Calculation of the yield of WJ-MSCs by 9 days post-WJ-MSC seeding. C: 5% CO_2_, 37 °C as a control, C+H: control + 5% O_2_ hypoxia, C+H+P: control + 5% O_2_ hypoxia + 2.0 PSI, mean ± S.E.M. * *p*-value < 0.05. Six batches per groups were assigned and tested.

In order to assess the proliferation and yield of the WJ-MSC primary culture, according to incubation conditions, cell confluency was observed by optical microscopy on the 4th, 7th, and 9th days; the cells were also collected on the 9th day in order to calculate yields (Figure 1). When the WJ-MSCs at passage 0 were exposed to standard incubation conditions (5% CO_2_, 37 °C incubator, as a control), or to the 5% O_2_ hypoxia conditions (C+H), or the 5% O_2_ hypoxia with 2.0 PSI pressure conditions (C+H+P), confluency changes were observed (Figure 1A). Since the primary culture (passage 0) was carried out using a cell mixture separated from umbilical cord tissue, a number of cells were generally observed on the 4th day of incubation. However, a relatively increased cell population was observed by the 7th and 9th day of incubation. Compared with the control group (standard incubation conditions), the initial adhesion of the cells was enhanced under the hypoxic, and hypoxia under high-pressure conditions, and the rate of cell adhesion was the highest under the hypoxia with high-pressure, conditions. When observing the cell proliferation up to the 9th day, the confluency was higher under the hypoxia with high pressure and the hypoxia conditions than under the control condition. Similarly, to the cell adhesion rates, cell proliferation under hypoxia with the high-pressure condition was most marked.

Next, the total yield of WJ-MSCs for each condition was measured on day 9 of incubation (Figure 1B). Under the control incubation condition, the initial yield was measured as 5.3 ± 0.9%. On the other hand, the initial yield was 12.0 ± 0.6% under the hypoxic condition, and the highest initial yield was found under the hypoxia with high-pressure conditions, at 18.0 ± 2.1%.

### 2.2. WJ-MSCs Shows the Highest Yield under 2.0 PSI Pressure Conditions

**Figure 2:**
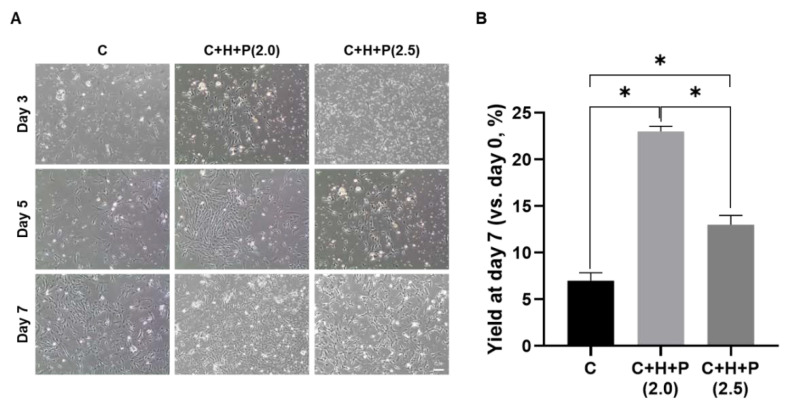
Confirmation of the yield of Wharton’s jelly-derived mesenchymal stem cells (WJ-MSCs) under different high-pressure conditions. (**A**) Observation of the shape of the cells through an optical microscope on the 3rd, 5th, and 7th days of the initial culture of the WJ-MSCs. The WJ-MSCs were cultured under control, 5% hypoxia with 2.0 PSI, or 5% hypoxia with 2.5 PSI pressure conditions. Scale bar = 100 µm. (**B**) Calculation of the yield of the WJ-MSCs by 7 days post WJ-MSC seeding. C: 5% CO_2_, 37 °C as a control; C+H+P (2.0): control + 5% O_2_ hypoxia + 2.0 PSI; C+H+P (2.5): control + 5% O_2_ hypoxia + 2.5 PSI. The data are presented as mean ± S.E.M. * *p*-value < 0.05. Six batches per groups were assigned and tested.

During the primary cultivation of the WJ-MSCs, we investigated the optimal pressure conditions under which the yield was maximized (Figure 2). The cells were initially cultured at different pressures (2.0 or 2.5 PSI) under 5% CO_2_, 37 °C, with 5% hypoxia, or under the standard incubation condition (5% CO_2_, 37 °C) as the control. After seeding on culture flakes, the morphology was observed using an optical microscope on the 3rd, 5th, and 7th days, and the cells were collected on the last (7th) day to calculate the yield. The WJ-MSCs’ yield, according to the incubation conditions, was observed by optical microscopy, and it was confirmed that the cells grew better under hypoxia with the high pressure condition, regardless of the pressure level, than under the standard incubation conditions (Figure 2A). When the 2.0 PSI and 2.5 PSI conditions are compared, the 2.0 PSI condition is more likely to promote cell growth than the 2.5 PSI condition. When the final yield of the cells for each incubation condition was calculated after 7 days of initial incubation, the results were similar to those obtained by optical microscopy (Figure 2B). A total cell yield of 7.0 ± 0.9% was calculated under the standard incubation condition, and was at its highest, at 23.0 ± 0.6%, under the 5% hypoxia and 2.0 PSI condition. The 2.5 PSI condition delivered a yield of 13.0± 1.0%, which was higher than that obtained under the standard incubation condition, but was less than that obtained under 2.0 PSI.

### 2.3. WJ-MSCs under High-Pressure Conditions Show Higher Cell Proliferation

**Figure 3:**
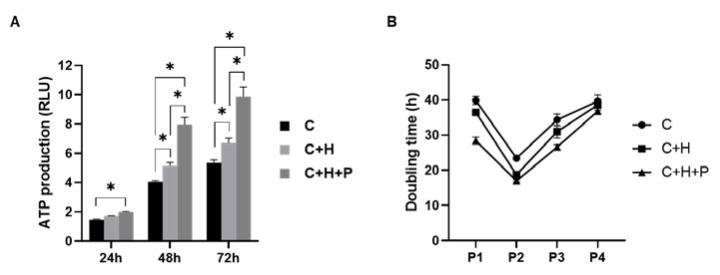
The viability and doubling time of the Wharton’s jelly-derived mesenchymal stem cells (WJ-MSCs) that were exposed to 2.0 PSI pressure with 5% hypoxic condition. (**A**) An ATP assay was performed in order to observe the cell viability of the WJ-MSCs at passage 0, at 24-, 48-, and 72-h post-initial cultivation under each condition. The data are presented as Mean ± S.E.M. (**B**) Doubling time of WJ-MSCs was calculated according to passage 1 and passage 2 of each condition. C: control; C + H: control + 5% O_2_ hypoxia; C+H+P: control + 5% O_2_ hypoxia + 2.0 PSI. * *p*-value < 0.05. Six batches per groups were assigned and tested.

The viability and doubling time of the primary WJ-MSCs, cultured under 2.0 PSI pressure with 5% hypoxia, were determined. An adenosine triphosphate (ATP) assay was performed at 24-, 48-, and 72-h post-initial cultivation under each incubation condition (Figure 3). The production of ATP under the high-pressure conditions with hypoxia showed the highest increase compared to the standard incubation or hypoxia-only conditions (Figure 3A). The difference between the three groups widened as the incubation time increased.

**Figure A1:**
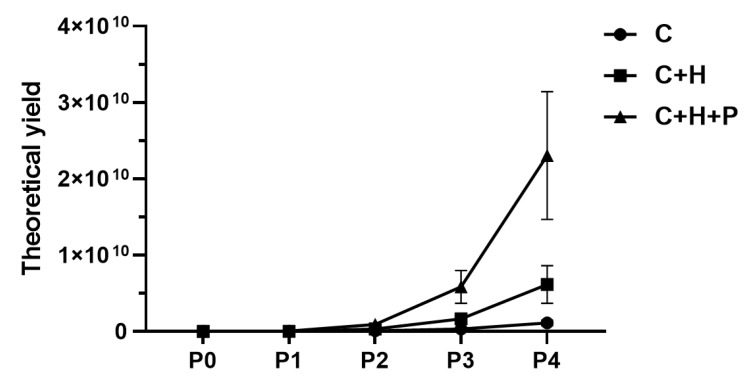
Theoretical yield of WJ-MSC at each passage. The theoretical yield of WJ-MSCs (C, C+H, and C+H+P) at each passage was calculated based on that of the doubling time.

Next, the doubling time of the cells was measured when the sub-culture was performed at passage 1 and passage 2, after the initial cultivation. In all three groups, there was a trend toward a decreased doubling time in passage 2, as compared to passage 1 (Figure 3B). Additionally, as with the ATP assay results, the MSCs cultured under conditions of high pressure showed the lowest doubling time among the three conditions, for all of the passages. The MSCs cultured under hypoxia also showed a lower doubling time than those cultured under the standard incubation conditions. Furthermore, the theoretical yields of the WJ-MSCs (C, C+H, and C+H+P) at passages 1 to 4 were calculated based on the doubling time (Appendix A Figure A1).

### 2.4. The Stemness of WJ-MSCs Is Maintained under High-Pressure Culture Conditions

**Figure 4:**
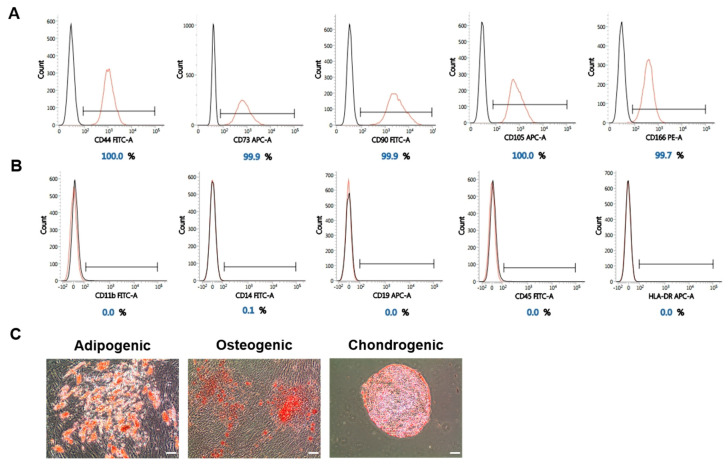
Stemness confirmation of Wharton’s jelly-derived mesenchymal stem cells (WJ-MSCs) exposed to the 5% hypoxia with 2.0 PSI pressure conditions. (**A**) The expression of MSC-positive markers (CD44, CD73, CD90, CD105, and CD166) was evaluated. (**B**) The expression of MSC-negative markers (CD11b, CD19, CD14, CD45, and HLA-DR) was evaluated. (**C**) WJ-MSCs were induced to differentiate into three-lineage cell types (adipogenic, osteogenic, and chondrogenic). Scale bar = 100 µm.

In the primary cultivation of WJ-MSCs separated from an umbilical cord, we confirmed that the culture of the cells under 5% hypoxia with 2.0 PSI pressure conditions facilitated the proliferation and increased the initial yield. The following immunophenotypic analysis and three-lineage differentiation were performed to confirm that WJ-MSCs exposed to the modified incubation conditions maintained their stemness (Figure 4). After passage 0, the WJ-MSCs were cultured step-by-step until passage 2, and an analysis was conducted using WJ-MSCs at passage 2. The analysis of the WJ-MSC surface marker was conducted first. Positive markers of MSCs were all found to be present in more than 95% (Figure 4A). We also confirmed that negative markers of MSCs were not expressed on the cells’ surface (Figure 4B).

Besides this, WJ-MSCs at passage 2 were induced to differentiate into a three-lineage cell type in order to evaluate their adipogenic, osteogenic, and chondrogenic characteristics, and a special staining was performed (Figure 4C). This confirmed that the MSCs which were initially cultivated under the 5% hypoxia with 2.0 PSI condition retained their differentiation potential.

### 2.5. Expression of Cell Proliferation-Related Genes Is Upregulated When WJ-MSCs Are Exposed to High-Pressure Conditions

**Figure 5:**
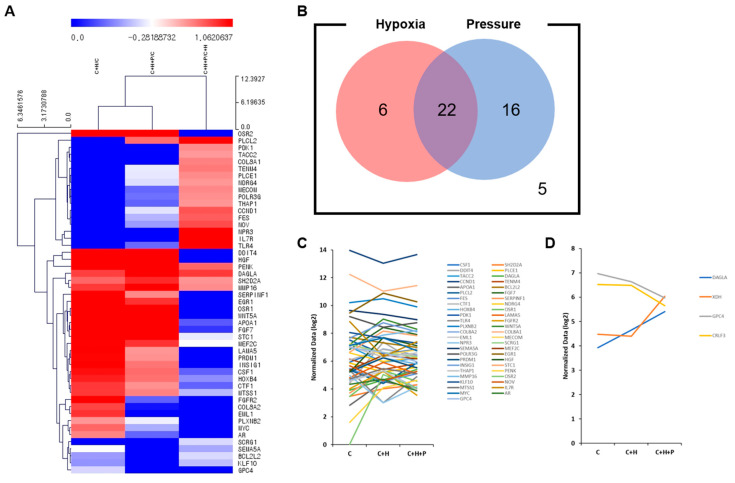
The 3′-mRNA sequencing analysis under high-pressure conditions. (**A**) Gene clustering analysis was conducted for 49 genes related to cell proliferation. The upregulated genes are represented in red, and the down-regulated genes are represented in blue. The name of each gene is shown to the right of the clustering map, and the groups or genes with similar expression patterns as a result of clustering were placed close together. (**B**) The genes upregulated upon exposure of the cells to high pressure and/or hypoxia are shown in the Venn diagram. The number of genes with increased expression under hypoxia only, under high pressure only, of under both conditions are shown inside of the circles, and the number of genes with decreased expression under these conditions is marked on the outside. (**C**) The mRNA expression change in the 49 selected genes, in relation to cell proliferation, is described as an expression plot. Under the C, C+H, and C+H+P conditions, the amount of the expression of each gene was normalized and converted to a log2 value. (**D**) *DAGLA* and *CRLF3* are related to cell proliferation; *GCP4* is related to the cell cycle; and *XDH* is related to therapeutic efficacy; these genes were selected and presented on the expression graph. C: control, C+H:+hypoxia, C+H+P: + hypoxia + pressure.

The 3′-mRNA sequencing was conducted in order to assess the difference in the mRNA expression of the WJ-MSCs at passage 2 (Figure 5). In the library, genes related to cell proliferation were selected according to their biological function classification. First, 49 genes of which expression was increased or decreased by 1.2 times were selected (Figure 5A). The C+H/C ratio indicates that the gene changed under hypoxic conditions, and the C+H+P/C ratio indicates that the gene changed under hypoxic and high-pressure conditions. The C+H+P/C+H ratio represents genes that changed expression under pressure conditions. Based on the fold-change values of these genes, gene clustering analysis confirmed that the C+H/C group was more similar to C+H+P/C group than to the C+H+P/C+H group. Next, for each selected gene, we classified and counted the number of genes depending on the hypoxia condition or the pressure condition, and these were described in a Venn diagram (Figure 5B). Consequently, we confirmed that there were six genes with increased expression only under the hypoxic conditions, 16 genes with increased expression only under the high-pressure conditions, and 22 genes with increased expression under both the hypoxic and high-pressure conditions. The mRNA expression of the five genes was decreased under both the hypoxic and high-pressure conditions. Next, the gene expression pattern for each experimental group was identified for the 49 genes related to cell proliferation (Figure 5C). The expression of each gene was normalized and converted to log2 values, and was plotted. *CCND1* was highly expressed in all three of the experimental groups overall, and *OSR2* was barely expressed under the standard cultivation conditions, but was upregulated when the WJ-MSCs were exposed to hypoxia and high pressure.

Among the 49 genes, *DALGA* and *GPC4*, which tended to show linear increases or decreases in expression under the hypoxia and high-pressure conditions, were selected as genes related to cell proliferation. *CRLF3* (related to the cell cycle) and *XDH* (related to the therapeutic efficacy of WJ-MSCs) were additionally selected with the same statistical criteria. These selected genes were represented on the expression graph (Figure 5D). The changes in the expression of these four genes noted under hypoxia, as compared to the standard incubation condition, were further exacerbated by high pressure.

### 2.6. The Anti-Apoptotic Effect of Wj-Mscs Is Maintained under High-Pressure Conditions

**Figure 6:**
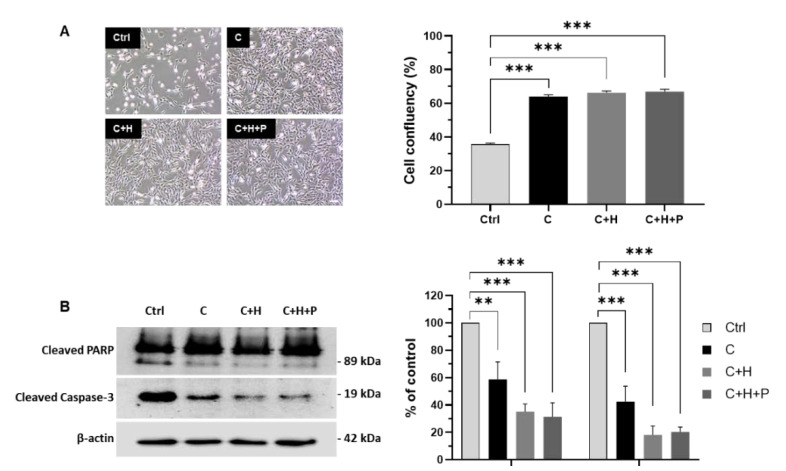
Therapeutic effects of WJ-MSCs on C2C12 cell death in an in vitro model. WJ-MSCs exposed to high-pressure and/or hypoxia were co-cultured with C2C12 cells in an in vitro cell death model. (**A**) After 24 h, microscopic images were taken of the C2C12 cells in each group, and their cell confluency was measured from these images. Scale bar: Scale bar = 100 µm. (**B**) The anti-apoptotic effects of WJ-MSCs on the C2C12 cells were confirmed by Western blot using cleaved PARP and cleaved caspase-3 antibodies. The bands were normalized to beta actin. The band intensities were quantified using Image J software. Control: C2C12 cells; C: co-cultured with control WJ-MSCs. C+H: co-cultured with WJ-MSCs exposed to hypoxia; C+H+P: co-cultured with WJ-MSCs exposed to high pressure and hypoxia. ** *p*-value < 0.01, *** *p*-value < 0.001. Six batches per groups were assigned and tested.

The cell death of C2C12 cells co-cultured with WJ-MSCs exposed to high pressure was assessed in order to evaluate the therapeutic effect of WJ-MSCs (Figure 6). First, WJ-MSCs were cultured under the standard incubation condition (C), the hypoxia condition (C+H), or the hypoxia with high-pressure conditions (C+H+P), and were then co-cultured with C2C12 cells in an in vitro model of cell death for 24 h. After co-culturing for 24 h, microscopic images were taken in order to evaluate the promotion of C2C12 cell proliferation (Figure 6A). Compared to the control group (confluency: 35.8%), the C2C12 cell death in vitro model, and the C2C12 myoblasts co-cultured with WJ-MSCs (C, C+H, or C+H+P) showed a significant increase in cell proliferation (64.0, 66.2, and 66.8% of confluency, respectively). However, the differences among the C, C+H, and C+H+P experimental groups were not significant when the cell confluency was quantified.

Next, a Western blot analysis using C2C12 cell lysates was performed in order to confirm the anti-apoptotic effects of WJ-MSCs (Figure 6B). Compared to the control group, the C2C12 myoblasts co-cultured with WJ-MSCs showed a decreased expression of cell death markers. In particular, WJ-MSCs exposed to hypoxia and high-pressure conditions (C+H+P) showed the highest anti-apoptotic effect on the C2C12 cell death model (cleaved poly ADP-ribose polymerase and cleaved caspase-3 showed 0.31 and 0.2-fold changes). The WJ-MSCs cultured under the hypoxic conditions (C+H) also showed an anti-apoptotic effect (cleaved PARP and cleaved caspase-3 showed 0.35 and 0.18-fold change).

## 3. Discussion

The clinical demand for MSCs has necessitated the development of methods for the robust production of large quantities of high-quality MSCs [20]. The advantage of large-scale cultivation is that it minimizes the differences between the MSC therapeutics used for cell therapy for multiple patients in a clinical trial [21]. Scaling up cell expansion would require substantial advancements in conditioned media quality, culturing devices, and techniques [22,23,24,25]. The most important step for large-scale cultivation is to obtain a sufficient number of MSCs at the primary cultivation stage [25]. The primary MSCs that were incubated for 7–10 days under standard incubation conditions provided an initial yield of 5% of the original number of cells. Because most of the primarily-isolated cells are somatic cells, only a fraction are MSCs [26,27]. Therefore, long-term cultivation is inevitably required in order to provide sufficient MSCs for clinical use [28].

Thus, obtaining increased yields of MSCs in a short time is technically challenging. In order to overcome these bottlenecks, we investigated the use of modified culture conditions, and demonstrated that physical force, particularly mechanical pressure stimuli, improved the initial yields of MSCs without causing a loss of stemness or therapeutic efficacy, as assessed by their anti-apoptotic effects. When it comes to the therapeutic effect, we did not study the mechanism of action (MoA) of the WJ-MSCs stimulated by pressure on anti-apoptotic effects. In further studies, based on the mRNA sequencing data, we are planning to screen the predicted therapeutic candidates that are expected to show therapeutic potential on muscle disease, and investigate their MoA. Many studies have attempted to promote the proliferation of MSCs, by genetic modification, the application of scaffolds, and the modification of the cultivation conditions [29,30,31,32]. In particular, through genetic modification, the target genes related to cell proliferation or the cell cycle could be overexpressed [30]. Therefore, this could be a direct method for the promotion of cell proliferation. However, promoting the proliferation rate of the stem cells through genetic manipulation faces the issues of having a high cost and introducing complicated additional steps before large-scale production for clinical implementation. As an alternative to genetic modification, we introduced mechanical force in order to modify the stem cell’s proliferation. Compared to genetic manipulation, applying physical force to stem cells is very simple and reasonable. It can be simply applied to a typical large-scale production process, and once the system is set up, it can be easily operated.

There are various types of mechanical forces, such as tensile, compressive, shear, osmotic, and fluid stresses. Previous studies have shown that a mechanical stimulus can mimic the stem cell niche, i.e., the essential microenvironment in which stem cells reside [33,34,35,36]. In this study, we attempted to simulate the stem cell niche in an in vitro system in which we cultured WJ-MSCs under mechanical force in order to promote cell proliferation. Additionally, the most widely known method of mimicking the in vivo environment of stem cells is the use of hypoxic culture conditions [37]. In order to improve a method of reflecting the stem cell niche in WJ-MSC culture systems, we introduced high-pressure conditions in addition to hypoxia. When the pressure was applied to the WJ-MSCs under hypoxic conditions, the stem cell proliferation rate increased in the primary culture (Figure 1). Consequently, we confirmed that mechanical pressure stimuli can mimic the stem cell niche and ultimately control the stem cells’ fate.

Argentati et al. reported that the extracellular matrix (ECM) plays a critical role in recognizing external physical forces [33,35]. The ECM interacts with stem cells in order to regulate cells’ fates, such as by promoting the proliferation or differentiation of the cells [38,39,40]. In addition, the cells can remodel the ECM [35]. In particular, the pressure used in this study is a type of compression, and the fate of cells is determined by the regulation of the Wnt/β-catenin signaling pathway after the recognition of compression through ECM components, such as collagen [33,41] and vimentin [42]. When it comes to genes that are significantly regulated by hypoxia and pressure, in particular, GPC4 decreased in both hypoxia and high-pressure conditions. Previous studies have shown that the Wnt pathway was promoted in the GPC4 overexpressed cell line [43]; therefore, it is expected that the decrease in the expression of GPC4 in MSCs exposed to hypoxia and pressure would inhibit the Wnt signing pathways. In addition, Wnt suppresses the proliferation of MSCs [44,45]. Although this study has not been experimentally confirmed, it is expected that the reduction in GPC4 expression plays a role in promoting the proliferation of MSCs by controlling Wnt signing through the interaction with the ECM.

In addition to GPC4, we found that the changing expression patterns remain consistent in both the hypoxia and pressure conditions for DAGLA [46] and CRLF4 [47]. It is not known whether high-pressure conditions show boosting or synergistic effects on genes’ expression in addition to the hypoxia condition, but further research is required, as there has been no previous study in relation to the proliferation of MSCs. WJ-MSCs exposed to pressure stimuli were demonstrated to maintain stemness, but to have a reduced doubling time compared to those exposed to standard incubation conditions (Figure 3 and Figure 4). Furthermore, we confirmed that the effect of promoting cell growth was maintained even when long-term cultivation was carried out under standard incubation conditions (Figure 3B). These data imply that these culture conditions can be used to produce a sufficient number of WJ-MSCs for clinical doses in a shorter time. Moreover, when we calculated the cumulative population doublings, based on the doubling time measurement, we confirmed that more than 20 times the MSC drug product (passage 4) could be produced by implementing pressure stimuli (Figure A1). However, in terms of genomic stability, these enhanced-proliferation MSCs might have a higher rate of mutation and an increased risk of tumorigenesis. However, in this study, the primary culture condition (passage 0) of WJ-MSC was applied with hypoxia or high-pressure conditions, and the general standard incubation conditions were applied for the subsequent cultivation of the WJ-MSCs.

As found in Section 2.4, the stemness and purity of the MSC remained up to two generations after the primary culture, and the WJ-MSCs were properly differentiated in their adipogenic, osteogenic, and chondrogenic natures. These results do not indicate an increase in tumorigenesis or the heterogeneity of WJ-MSCs due to the genetic mutation caused by the modified culture conditions. In addition, when we observed the doubling time according to the subsequent culture up to passage 4 from Section 2.3, we found that the doubling time of C and C+H+P was significantly different in passage 0, while passage 4 showed little difference in the doubling time between the C and C+H+P experimental groups. This finding indicates that the increase in the cultivation time in standard conditions after the primary culture will dilute the proliferation improvement effect due to hypoxia or high pressure. Therefore, it can be concluded that the growth rate improvement under exposure to hypoxia and high-pressure conditions during the primary culture is not an irreversible change.

However, the safety issue of WJ-MSCs exposed to hypoxia and high-pressure could be evaluated using a karyotype analysis or a teratoma assay in a further study. Generally, due to safety issues, xenogeneic origin reagents, such as fetal bovine serum (FBS), must be removed by multiple washing steps at the final stage of production for clinical use. As an alternative to multiple washing stages, a xeno-free culture condition has been recently developed. However, we chose multiple steps of washing for the production of clinical-grade stem cells. Instead, we screened FBS (Gibco, Cat. No.: 16000-044, Gibco, Waltham, MA, USA) for bovine-derived viruses (e.g., bovine viral diarrhea virus) according to EMA, FDA, and MFDS guidelines for Cell therapeutics. In addition, in order to remove the remaining FBS components, we performed multiple cleaning steps using PBS in the final stage. Finally, the GMP facility at Samsung Medical Center checked the remaining bovine serum albumin (BSA) and gentamicin concentration by ELISA analysis during the quality control (QC) stage.

In conclusion, our results suggest that mechanical pressure stimuli play a key role in improving the initial yields of MSCs. In particular, mechanical cues to maintain and expand MSC populations in the culture are potentially important for scaling up the production of these cells for therapeutic applications. In addition, a combination of techniques, including biomaterials modified with growth factors or ligands, as well as other growth parameters for optimal growth, would be essential in order to advance the fields of cell therapy, tissue engineering, and regenerative medicine.

## 4. Materials and Methods

### 4.1. Isolation and Cultivation of Human Wharton’s Jelly-Derived Mscs under Normal Conditions

This study was approved by the institutional review board of the Samsung Medical Center (IRB#2016-07-102). The umbilical cords were collected with informed consent from pregnant mothers. From the umbilical cord tissues, Wharton’s jelly-derived mesenchymal stem cells (WJ-MSCs) were isolated, and the primary culture was performed according to the procedure of Kwon et al. [48], and the standard operating procedures of the Good Manufacturing Practice facility at the Samsung Medical Center.

The umbilical cord tissues used in this study were donated from six donors. The umbilical cords were washed thoroughly to remove the blood, and were then cut into 1.5-cm-long pieces. In order to isolate cells from Wharton’s jelly, each piece was then cut open lengthwise with sterile scissors and forceps, and the umbilical blood vessels were removed. The gelatinous tissue surrounding the vessels was excised and minced into fine pieces, and placed in sterile 50 mL centrifuge tubes in a 0.2% collagenase type I solution. After 40 min, an equal volume of MEMα (Gibco, Waltham, MA, USA) with fetal bovine serum (FBS; Gibco) was added, and the samples were centrifuged at 300× *g* for 10 min. The supernatant was discarded, and the cells were plated in a 75T flask. After isolation, each WJ-MSC was separated into the experimental groups (standard culture conditions, hypoxia conditions, and hypoxia+ 2.0 or 2.5 high-pressure conditions). Six batches of each experimental group were tested in this study.

In the case of the standard culture condition, WJ-MSCs plated in a 75T flask were cultured at 37 °C in a 5% CO_2_ incubator. Hypoxia and high-pressure conditions were applied to the primary cultures of WJ-MSCs (passage 0) using the Avatar^TM^ Cell Control System (Xcell Biosciences, Huntersville, NC, USA). The parameters were set as follows: hypoxia condition (5% CO_2_, 5% O_2_, and 37 °C temperature); 2.0 PSI high-pressure condition: 5% CO_2_, 5% O_2_, 7.2–7.5 pH, 37 °C temperature, and 2.0 PSI); and 2.5 PSI high-pressure condition (5% CO_2_, 5% O_2_, 7.2–7.5 pH, 37 °C temperature, and 2.5 PSI).

The WJ-MSCs were expanded in MEMα medium (Gibco) containing 10% FBS (Gibco) and 50 ug/mL gentamicin (Gibco), and were sub-cultured to passage 5 under standard conditions of 5% CO_2_ at 37 °C.

### 4.2. Calculation of the Wj-Mscs Yield at the Primary Culture

A cell mixture containing WJ-MSCs separated from the Wharton’s jelly was seeded with a concentration of 50.000–100,000 cells/cm^2^ (total 2.5–7.5 × 10^5^ cells) in a 75T flask, as described in Materials and Methods 4.1. After 7 or 9 days of incubation, we collected all of the remaining cells in a 75T flask and counted them. The parameter ‘yield’ was calculated as follows: the total number of the harvested cells after 9 days of incubation was divided by the number of cells initially seeded (2.5–7.5 × 10^5^), and was reported as a percentage.

### 4.3. Characterization of Hypoxia- and High-Pressure-Cultured Wj-Mscs

The WJ-MSCs (passage 2) were detached using 0.25% EDTA-Trypsin solution, and were harvested in a 15-mL conical tube. After centrifugation, the WJ-MSCs were washed and resuspended in phosphate-buffered saline with 2% FBS to block the nonspecific binding sites. According to the MSC criteria of the International Society for Cell Therapy [2], an immunophenotypic analysis of the WJ-MSCs was performed via flow cytometry analysis, using the following markers: CD11b, CD14, CD19, CD44, CD45, CD73, CD90, CD105, CD166, and HLA-DR (BD Biosciences, Franklin Lakes, NJ, USA). At least 10.000 events were acquired on a BD FACSVerse (BD Biosciences), and the results were analyzed with BD FACSuite software v.10 (BD Biosciences).

The differentiation of the WJ-MSCs was tested according to the procedure outlined in a previous report [49].

### 4.4. Cell Proliferation Assay

A cell proliferation assay was performed on the WJ-MSCs at passage 0. At 24, 48, and 72 h of the WJ-MSCs’ primary culture, the amount of ATP produced was measured using the CellTiter-Glo^®^ kit (Promega, Madison, WI, USA), following the manufacturer’s instructions.

### 4.5. RNA Isolation

The total RNA was isolated using TRIzol reagent (Invitrogen, Carlsbad, CA, USA). The RNA’s quality was assessed by means of an Agilent 2100 bioanalyzer using the RNA 6000 Nano Chip (Agilent Technologies, Amstelveen, The Netherlands), and the RNA’s quantification was performed using an ND-2000 Spectrophotometer (Thermo Inc., Waltham, MA, USA).

### 4.6. Library Preparation and Quantseq 3′ mRNA Sequencing

The library construction of the control and test RNAs was performed using a QuantSeq 3′ mRNA-Seq Library Prep Kit (Lexogen, Inc., Vienna, Austria) according to the manufacturer’s instructions. In brief, 500 ng of the total RNA sample was prepared, and an oligo-dT primer containing an Illumina-compatible sequence at its 5′-end was hybridized to the RNA, after which a reverse transcription was performed. After the degradation of the RNA template, second-strand synthesis was initiated by a random primer containing an Illumina-compatible linker sequence at its 5′-end. The double-stranded library was purified using magnetic beads in order to remove all of the reaction components. The library was amplified in order to add the complete adapter sequences required for the cluster generation. The finished library was purified from the PCR components. High-throughput sequencing was performed via single-end 75 sequencing using NextSeq 500 (Illumina, Inc., San Diego, CA, USA).

### 4.7. QuantSeq 3′ mRNA Sequencing Data Analysis

The QuantSeq 3′ mRNA-Seq reads were aligned using Bowtie2 [50]. Bowtie2 indices were either generated from the genome assembly sequence or the representative transcript sequences. The alignment file was used to assemble the transcripts, estimate their abundances, and detect the differential expression of the genes. The differentially-expressed genes were determined based on the unique counts and multiple alignments covered in Bedtools [51]. The RT (Read Count) data were processed based on the Quantile–Quantile normalization method using EdgeR within R [52], using a Bioconductor [53]. The gene classification was based on searches conducted in the DAVID (http://david.abcc.ncifcrf.gov/) and Medline databases (http://www.ncbi.nlm.nih.gov/).

### 4.8. C2C12 Cells Culture

The mouse immortalized myoblast cell line, C2C12 (ATCC CRL-1772, American Type Culture Collection, Rockville, MD) was cultured in Dulbecco’s Modified Eagle’s medium (Biowest S.A.S, Nuaille, France) supplemented with 10% FBS (Gibco BRL, Carlsbad, CA), 100 U/mL penicillin, and 100 µg/mL streptomycin (Gibco BRL) in 5% CO_2_ at 37 °C.

### 4.9. Induction of Cell Death in C2C12 Cells and Co-Culture with Wj-Mscs

C2C12 cell death was induced in vitro, according to a previous study [48]. After thawing and maintaining the cell line, C2C12 cells were seeded in 6-well plates with 1 × 10^5^ cells/well. After 24 h, cell death was induced in C2C12 cells by treatment with 10 µ M lovastatin. On the day of initiating the co-culture, 1 × 10^5^ WJ-MSCs (C, C+H, and C+H+P) were detached and seeded on the 6-well insert chamber, and were directly transferred to the 6-well plates in which C2C12 cells were cultivated. The co-culture was maintained for 24 h, and all of the C2C12 cells were harvested for Western blot analysis.

### 4.10. Western Blot Analysis

The C2C12 cell extracts described in Section 4.7 were prepared by ultrasonication (Branson Ultrasonics Corporation, Danbury, CT, USA) using a buffer containing 9.8 M urea, 4% CHAPS (3-[(3-Cholamidopropyl)dimethylammonio]-1-propanesulfonate), 130 mM dithiothreitol, 40mM Tris-HCl, 0.1% sodium dodecyl sulfate, 1mM EDTA (Ethylenediaminetetraacetic acid), and a protease/phosphatase inhibitor cocktail. A protein quantification was performed using the Bradford assay (Bio-Rad Laboratories, Hercules, CA, USA). The protein extracts (10–20 µg) were separated by SDS-PAGE (Sodium Dodecyl Sulfate Polyacrylamide Gel Electrophoresis), and the resolved proteins were transferred to polyvinylidene fluoride membranes. Each membrane was incubated with the above-mentioned antibodies. The membranes were blocked with 5% skim milk in TBS-T (containing 0.2% Tween-20) at room temperature with gentle shaking for 60 min, followed by incubation with primary antibodies (1:1000–1:5000 dilutions in 5% skim milk) for 1–1.5 h at room temperature or overnight (4 °C), with gentle shaking. After washing in TBS-T (3×, 10 min/wash), the membranes were incubated with HRP-conjugated anti-rabbit or anti-mouse secondary antibodies (1:5000–1:10,000 dilutions in 5% skim milk) at room temperature for 1 h, with gentle shaking. The membranes were washed (3×, 10 min/wash) and treated with an enhanced chemiluminescence solution (Advansta Inc., San Jose, CA, USA) for 30 s before detecting the bands through exposure to film.

### 4.11. Antibodies and Reagents

The following primary antibodies were used for the experiment: Poly ADP ribose polymerase PARP (Cell Signalling Technology, Danvers, MA, USA), Cleaved Caspase 3 (Cell Signalling Technology, Danvers, MA, USA), and Beta-actin (Santa Cruz Biotechnology, Dallas, TX, USA).

### 4.12. Statistical Analyses

All of the values are presented as the mean ± standard error of the mean (S.E.M). One-way ANOVA was used to assess significance, and a *p*-value ≤ 0.05 was considered statistically significant. IBM SPSS software, v. 21.0, was used for all of the analyses.

## Abbreviations

ECM	Extracellular matrix
FBS	Fetal bovine serum
MSC	Mesenchymal stem cell
WJ	Wharton’s jelly
WJ-MSCs	Wharton’s jelly-derived mesenchymal stem cells
PCR	Polymerase chain reaction
GPC4	Glypican 4
DAGLA	Diacylglycerol lipase alpha
CRLF3	Cytokine receptor like factor 3
XDH	Xanthine dehydrogenase
CCND1	Cyclin D1
OSR2	Protein odd skipped related 2
ELISA	Enzyme linked immunosorbent assay
PSI	Pounds per square inch
ATP	Adenosine triphosphate
mRNA	Messenger ribonucleic acid
PARP	Poly (ADP-ribose) polymerase
HIF-1	Hypoxia-inducible factor 1
S.E.M.	Standard error of the mean
BSA	Bovine serum albumin
EMA	European medicines agency
FDA	Food and drug administration
MFDS	Ministry of food and drug safety
GMP	Good manufacturing practice
QC	Quality control
